# Supplementary material for: Neuroimaging evidence for a network sampling theory of individual differences in human intelligence test performance
Source: Nat Commun. 2021 Apr 6;12:2072. doi: 10.1038/s41467-021-22199-9 (PMC8024400; doi:10.1038/s41467-021-22199-9)
Supplement: Supplementary file 1 — Supplementary Information [file 41467_2021_22199_MOESM1_ESM.pdf]

Supplementary Information for

Neuroimaging evidence for a network sampling theory of individual differences in human intelligence

Eyal Soreq,Ines R. Violante,Richard Daws,Adam Hampshire

eyal.soreq@ukdri.ac.uk

This PDF file includes:

Supplementary Figure 1 to 7
Supplementary Tables 1 to 5
SI References

Supplementary Methods

A. Supplementary Material

A.1. Behavioural Tasks

The twelve cognitive tasks (Figure 1) were conceived, designed and programmed by A.H., based on well-established paradigms from the cognitive neuroscience literature, to measure planning, reasoning, attention, and working memory abilities. They form the basis of multiple previous publications and are briefly described below (see supplement movies for additional visualisation of the tasks). While the neuroimaging version of the tasks did not have any learning feedback, the web version provided participants with a score that reflected performance. Both versions were adaptive to the participant's individual performance by increasing the task difficulty using a step function. The outcome measures are taken from the supplementary material of (1).

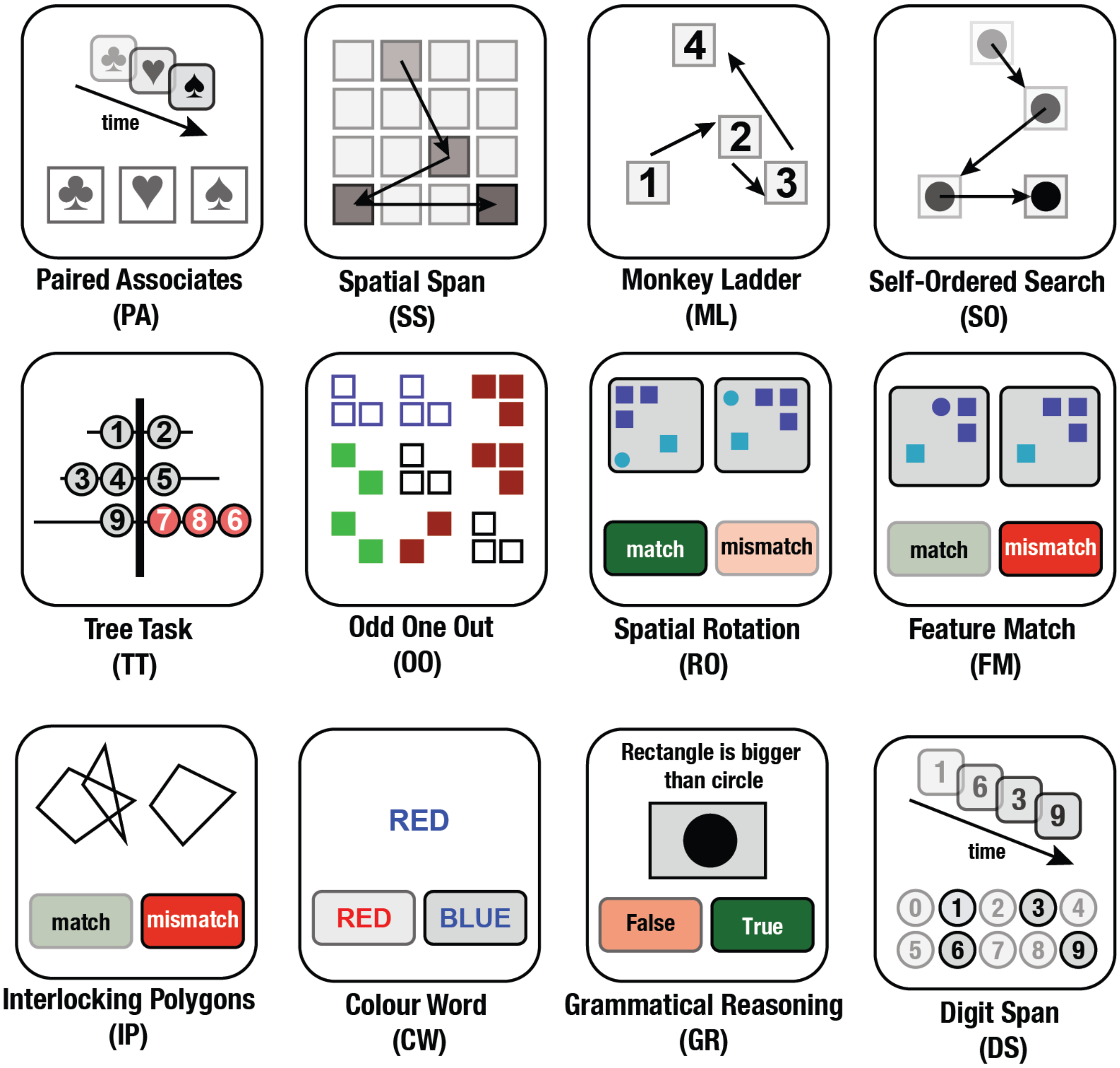

SUPPLEMENTARY FIGURE 1. The intelligence battery used in this study includes 12 different tasks covering a mixture of cognitive abilities considered to be central for intelligent behaviour. The same tasks were used in both the fMRI study and online web studies. The toy illustration attempt to capture the critical aspects of each task and allow to compare across tasks. More in-depth description can be found in the following text and the supplementary movies. The movies capture actual one-minute interaction of each fMRI variant of the tasks combined with saliency analysis.

A.2. Behavioural scores

SUPPLEMENTARY TABLE 1. Within scanner behavioural scores.

Task name	ID	Performance (std)	Task name	ID	Performance (std)
Paired Associates	PA	3.9107 (0.474)	Spatial Span	SS	5.2369 (0.8807)
Monkey Ladder	ML	5.483 (0.4657)	Self-Ordered Search	SO	7.95 (1.64)
Tree Task	TT	24.483 (7.7645)	Odd one out	OO	13.3 (2.8422)
Spatial Rotation	RO	205.3 (92.68)	Feature Match	FM	284.71 (78.968)
Interlocking Polygons	IP	99.35 (44.956)	Colour Word	CW	78.567 (18.341)
Grammatical Reasoning	GR	34.35 (9.3769)	Digit Span	DS	5.7686 (0.68378)

A.3. Task description

Paired Associates (PA) - This task is based on a paradigm commonly used to assess memory impairments in ageing clinical populations(2). Boxes are displayed at random locations on an invisible 5*5 grid. The boxes open one after another to reveal an enclosed object. This is followed by displaying the objects in the centre of the grid in random order. The participant is required to click on the boxes that contained each object. The difficulty is adjusted by increasing/decreasing the number of object-box pairs by 1. Difficulty increases if all pairs are correctly recollected. Outcome measure is calculated as the maximum level achieved (max=24,min=2 Population (mean,sd) = 5.28 ± 1.13).

Spatial Span (SS) - Based on the Corsi Block Tapping Task(3), which is a classical task for measuring spatial short-term memory capacity. 16 squares are displayed in a 4*4 grid. A sub-set of these squares flashes in a random sequence (1 flash every 900 ms). The participant is required to repeat the sequence by clicking on the squares in the same order in which they were flashed. The difficulty is dynamically varied based on accuracy, such that if the sequence is performed correctly the length of the next sequence is increased by one flash, otherwise, the sequence is one flash shorter. Outcome measure is calculated as the maximum level achieved (max=16,min=2 Population (mean,sd) = 6.15 ± 1.07).

Monkey Ladder (ML) - Visuospatial working memory task based on the non-human primate literature(4). Sets of numbered squares are displayed at random locations within an invisible 5*5 grid. After a variable interval (900ms * number of squares), the numbers are removed while the squares are kept in the screen. Participants are required to click the squares in ascending numerical sequence. The difficulty of the task increased or decreased by 1 square depending on the accuracy of the response. Outcome measure is calculated as the maximum level achieved (max=25,min=2 population (mean,sd) = 7.85 ± 1.154).

Self-Ordered Search (SO) - Based on a test used to measure strategy during search behaviour(5). Sets of boxes are displayed in random locations within an invisible 5*5 grid. The participant is required to find a hidden 'token' by clicking on a box at a time to reveal its contents. Once the token is found it is hidden in another box. On any given trial, the token is only placed in a box once, forcing the participant to search all boxes until the token has been found once in each box. If the participant clicks on the same box twice whilst looking for the token or searches a box in which the token has previously been found, this is an error and the trial ends. In this case, a new trial begins with one less box to search. If no errors are made, a new trial begins with one extra box. Outcome measure is calculated as the maximum level achieved (max=25,min=2 Population (mean,sd) = 8.23 ± 2.1).

Tree Task (TT) - Spatial planning task based on the Tower of London Task(6), which is widely used to measure executive function. Beads with numbers are positioned on a tree-shaped frame. The participant is required to position the beads in ascending numerical order from left to right and top to bottom. The participant must solve as many trials as possible within the duration of the block. The difficulty is increased by increasing the number of beads and planning complexity in increasing steps. Trials are aborted if the participant makes more than twice the number of moves required to solve the problem. Outcome measures = total score. Population (mean,sd) = 64 ± 10.185.

Odd one out (OO) - A deductive reasoning task based on a sub-set of problems from the Cattell Culture Fair Intelligence Test(7). A 3*3 grid of cells is displayed on the screen, containing a varied number of copies of a particular shape. The features that make up the objects in each cell (colour, shape, number of copies) are related to each other according to a set of rules. The participant is required to deduce the rules that relate the object features and select the cell whose contents do not match the rules. If the sequence is correct the problem increases in complexity. Outcome measure = total correct. Population (mean,sd) = 10.43 ± 3.31.

Spatial Rotation (RO) - Tasks of this type are typically used to measure the ability to manipulate objects in mind(8). Two grids of coloured squares are displayed side by side rotated by a multiple of 90 degrees. When rotated, the grids are either identical or differ by the position of one square. The participant is asked to indicate whether the grids are identical. If the response is correct the number of squares increases and if it is incorrect the number of squares decreases. Outcome measure = total score. Population (mean,sd) = 88.72 ± 36.32.

Feature Match (FM) - Based on classic feature search tasks that have been historically used to measure attentional processing(9). Two grids are displayed, each containing a set of abstract shapes. In half of the trials, the grids differ by just one shape. The participant is required to indicate whether the grid's contents are identical. If a trial is correct the total number of shapes increases, if it is incorrect the number of shapes is reduced. Outcome measure = total score. Population (mean,sd) = 131.35 ± 32.79.

Interlocking Polygons (IP) - Based on the Interlocking Pentagons task, which is often used in the assessment of age-related disorders(10). A pair of polygons is displayed on one side of the screen. The participant indicates whether a polygon displayed on the other side of the screen is identical to one of the interlocking polygons. If responses are correct the differences between polygons become increasingly subtle. If the response is incorrect the differences between polygons become more pronounced. Main outcome measure = total score. Population (mean,sd) = 51.41 ± 24.86.

Colour Word (CW) - This is a more challenging variant on the Stroop test(11). A coloured word is displayed at the top of the screen. For example, the word RED drawn in blue ink. The participant indicates which of two coloured words at the bottom of the screen described the colour of the word at the top of the screen. The colour word mappings may be congruent, in-congruent, or doubly in-congruent, depending on whether the colour that a given word describes matches the colour of the ink. The participant solves as many problems as possible within the duration of the block. Outcome measure = total score. Population (mean,sd) = 30.92 ± 13.01.

Grammatical Reasoning (GR) - This is a verbal reasoning task based on Alan Baddeley's 3-minute grammatical reasoning test(12). Problems of the form "The square is not encapsulated by the circle" are displayed on the screen and the participant indicates whether the statement correctly describes the pair of objects presented. Outcome measure is calculated as the maximum level achieved (max=25,min=2 Population (mean,sd) = 17.38 ± 5.01).

Digit Span (DS) - Is a computerised variant on the verbal working memory component of the WAIS-R intelligence test(13). Participants view a sequence of digits that appear one after another. Subsequently, they repeat the sequence of numbers by clicking on the corresponding digit on a keyboard displayed on the screen. The difficulty is dynamically varied by increasing or decreasing the number of digits to remember by 1, depending on whether the participant got the previous trial correct. Outcome measure is calculated as the maximum level achieved (max=25,min=2 Population (mean,sd) $= 7.22 \pm 1.52$).

A.4. Dynamic saliency

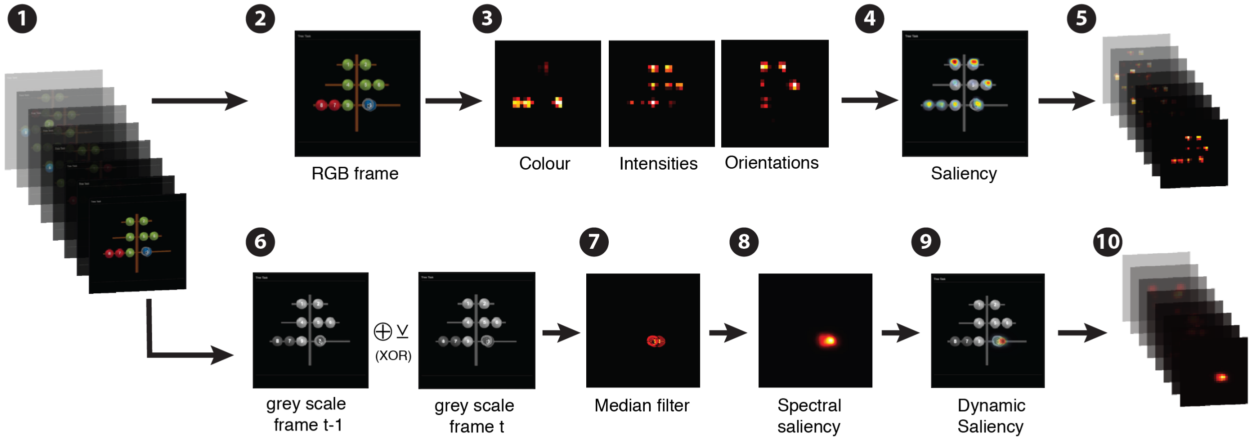

SUPPLEMENTARY FIGURE 2. Dynamic Visual saliency Approximation
A minute of task interactions was video captured for each of the 12 tasks, all frames were then loaded onto an (1) 4d matrix composed of 1800 consecutive frames (30 frames * 60 seconds) (2) for each colour frame centre-surround saliency is estimated (3) The model estimates colour, intensity and orientation saliency independently (4) which is them combined to form the final static saliency map (5) the process is performed for each of the t frames. (6) The logical exclusive OR (xor) on two grey scale consecutive frames (capturing only the bits that different in either frames) is used as a mask to capture only pixels that are changing across time. (7) An aggressive 20 x 20 median is applied to remove noise. (8) Spectral saliency is used to estimate (9) the final saliency map. (10) the same process is applied to all consecutive frame pairs.

During the revision stage, an important point was raised relating to the fact that each of the tasks has a distinct temporal pattern that is driving mostly by the tasks output and input mechanics (i.e. the timing of stimuli presentation and the type of motor response the task demands). As a result, it was proposed by one of the reviewers, that behavioural and Bold based similarities across tasks are driven by the similarities within these temporal patterns. If as proposed, these temporal patterns are driving the similarities between task, then composite scores that capture the temporal attention load of stimuli and response dynamics should resemble the task clusters that are evident in both the neuronal and behavioural measures. While we were unable to recreate in retrospect the actual task sequence every person played, we were able to measure the saliency patterns(14) from a representative minute of interaction from each task. Visual saliency models are based on visual perception theory and try to predict human fixations regions of interest or identifying the salient regions from mostly natural imagery or video to assist in object segmentation(15). In this study, we used bottom-up saliency models to capture the pairwise temporal similarity between tasks and how it relates to other measures of similarity explored here. Our two saliency models rely on two different established models(16, 17); the first used to capture static saliency by combining centre-surround models based on colour, intensity and orientation information from each frame independently(16) (Figure 22-5). And the second captures the dynamic temporal change by estimating the spectral residual (using Fourier transform) of two consecutive frames(17) (Figure 26-10). The first measure captures the relative attention load per frame. In contrast, the latter detects the dominant dynamic events (i.e. appearance and disappearance of stimuli as well as motor response to task demands). As can be seen in the 12 (per task) supplementary movies, these measures successfully

capture the differences in block structure across tasks, as well as the inner dynamics each task has. Importantly, the goal here was to build on these established models to generate a metric that reflected motor interactions dynamics and differences in visual attention load for each task.

After calculating both saliency matrices (for each task), we simply sum the scores across pixels to form two independent per frame saliency composite scores. Scores are then normalised (to a range of 0-1). The mean of the summation of both scores is also examined (Figure 3 1-3). Person cross-correlations across these vectors are used as a similarity measure (Figure 3 4-6). Finally, the upper triangle of each of these matrices is compared to the psychometric similarity (Figure 3 7-9). Our results showed that all tasks have very different saliency temporal patterns and that these pairwise relationships are not significantly related to the psychometric similarity.

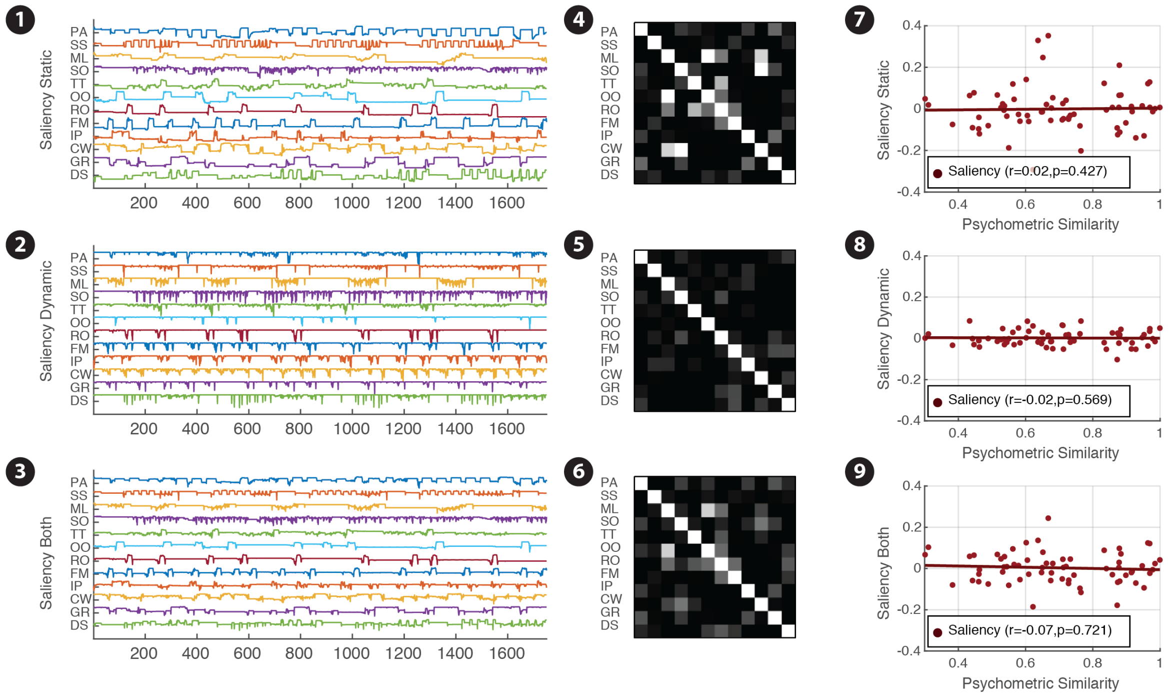

SUPPLEMENTARY FIGURE 3. Comparing saliency measures to psychometric similarity
(1-3) Saliency composite scores across tasks per frames (1. Static, 2. Dynamic 3. Combination) (4-6) Task pairwise Pearson cross-correlation matrices for the different saliency measures. (7-9) Scatter plots showing the relationship between the different salience measures and behavioural psychometric similarity.

A.5. Task movies

To ensure maximum transparency, one minute of task engagement was video captured. Each task engagement video was then converted to individual frames and a visual dynamic saliency analysis was conducted and visualized. We supply these 12 movies as part of the supplementary information. Each supplementary movie is composed of five panels. The top two panels show summary scores (normalized sum per frame) from two complementary visual saliency approximation algorithms. The first from a gold standard centre surround Saliency detection toolbox(14) and the bottom one representing our own modification to the spectral Saliency model to capture large dynamic changes. The bottom three panels show captured task engagement, where in the right panel we superimpose the static centre surround Saliency model over the task stimuli and in the right panel we show the dynamic spectral saliency model. Visual inspection of the movies, as well as statistical analysis visualised in supplementary Figure 3 suggests that these tasks exhibit independent temporal patterns that reflect the differences in dynamic engagement between tasks. Fur-

thermore, it is clear that these are unrelated to the behavioural psychometric similarity extracted from the online study.

B. Neuroimaging methods

B.1. Pre-processing

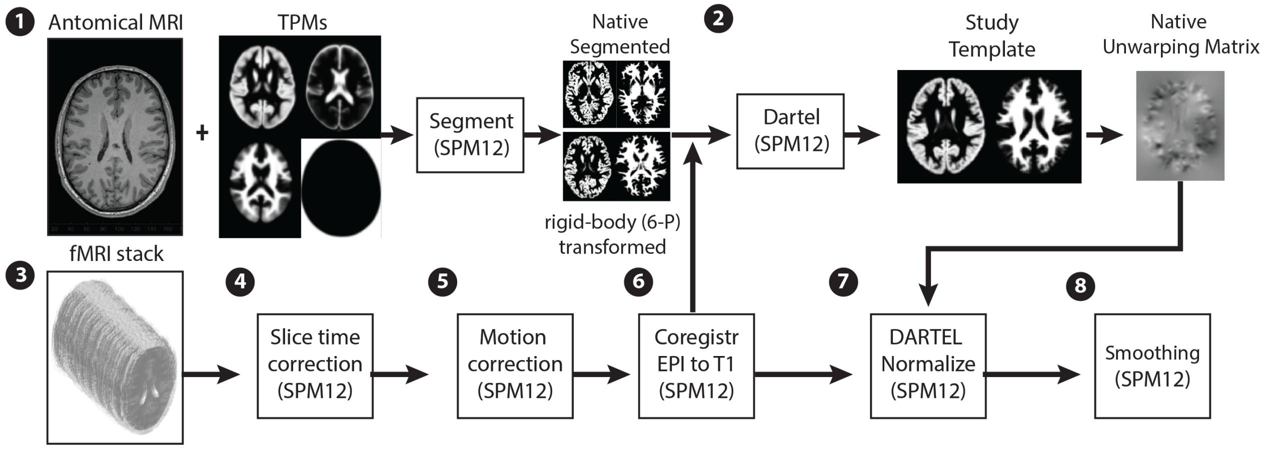

SUPPLEMENTARY FIGURE 4. pre-processing pipeline
The following pre-processing stages were performed: 1) for each individual structural segmentation is performed in native space using tissue probability maps 2) group DARTEL template was created from all subject's grey matter and white matter segmentation volumes 3) for each functional scan the first ten volumes were removed; 4) they were then slice-time corrected (using SPM12); 5) motion-corrected (SPM12); 6) epi volumes were coregistered to structural scans 7) spatially warped onto the standard Montreal Neurological Institute template using the custom DARTEL template generated at stage-2, 8) and spatially smoothed with an 8 mm3 full width at half maximum Gaussian kernel. 9) The data were high-pass filtered to remove low-frequency drifts. 10) Nuisance experimental matrices were formed using motion parameter estimates (in an extended 24-parameter model) and motion scrubbing (spikes) events estimated using frame-wise displacement (FSL5.0) FD=0.5.

All functional and anatomical scans were preprocessed using a custom pipeline including SPM12 (Statistical Parametric Mapping Welcome Department of Imaging Neuroscience), FSL (FMRIB Software Library v5.0) and MATLAB 2016b (Figure 4). The pipeline begins by forming a group structural template using the non-linear DARTEL(18) algorithm that is based on the tissue specific segmentation of each T1 map. Then all 12 fMRI 4D volumes are slice-time corrected (using an interleaved order) with the first slice acting as reference. This is followed by a realignment stage where for each subject all the first volumes in all sessions were realigned to the first volume in the first session. Then volumes within each session were aligned to the first image of the first session (quality=1, separation=5mm, smoothing=5, and 7 degrees of interpolation). A by-product of this stage are the realignment parameters that indicate the per volume displacement from the first volume in each session. The volumes are then co-registered to the native T1 structural volume, by registering the mean EPI volume to the T1 volume and applying the affine registration parameters to all 12 4D volumes. Finally, all resliced volumes are transformed onto MNI space using the DARTEL normalisation pipeline which includes interpolating the data to 2 x 2 x 2 mm voxel size and smoothing with a 8 mm3 full width at half maximum Gaussian kernel. Raw EPI volumes are also used to estimate spiking events using frame-wise displacement (FSL5.0) with a threshold value of FD=0.5.

B.2. Motion and noise correction

To account for the impacts of motion artefacts in both activation and connectivity estimate we used the approach recommended by Yan et al. (19) incorporated in the design matrix of the different metrics. Specifically we used (1) the friston 24-parameter(20) Higher-order regression model that regress out 6 head motion parameters, their derivative, and the 12 corresponding squared items. (2) Scrubbing using frame wise displacement(21) to identify spike time points using a threshold of FD>0.5 mm and then modelling each spike as a separate regressor in the regression models. Following comments from the revision process, in the case of connectivity only we also used tissue segmentation's to extract tissue specific mean EPI signal as well as a global brain mean signal. All time series are demeaned and de-trended and the 4 time-series (i.e. grey and white matter CSF and global signal) and their derivatives are added as a separate regressor in the regression models.

B.3. Statistical Parametric Mapping (i.e. individual activation estimate)

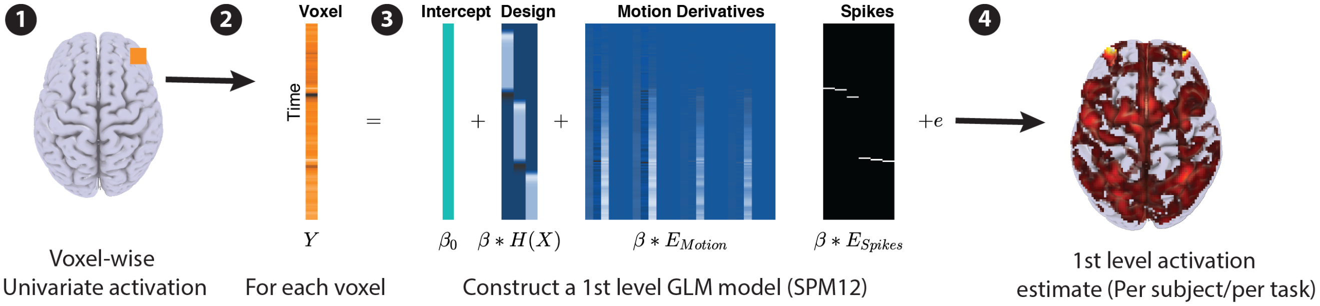

SUPPLEMENTARY FIGURE 5. Mass univariate generalized linear model (GLM) model
Using the SPM12 GLM mass univariate GLM pipeline, (1) each voxel time-series is independently modelled using a regression model (3) containing an intercept for each volume, a block design matrix convolved with the canonical HRF, the Friston 24 motion derivatives and the identified spikes to form per TR bold activation estimate. level task activation's were estimated against the 20s inter-block resting state baseline (4) to form a per task per subject activation estimate a contrast is used to sum block beta coefficients together across run replication (for each voxel).

A per participant unique design matrix was constructed from the HRF convolved experimental onsets and matrix of nuisance variables. This was applied to the fMRI data to estimate beta coefficient fits per voxel using the classic mass-univariate GLM in SPM12. Lastly, using predefined contrasts of interest, whole-brain maps depicting statistical parametric estimates were generated for each block and for each task.

B.4. Dynamic functional connectivity FIR estimation

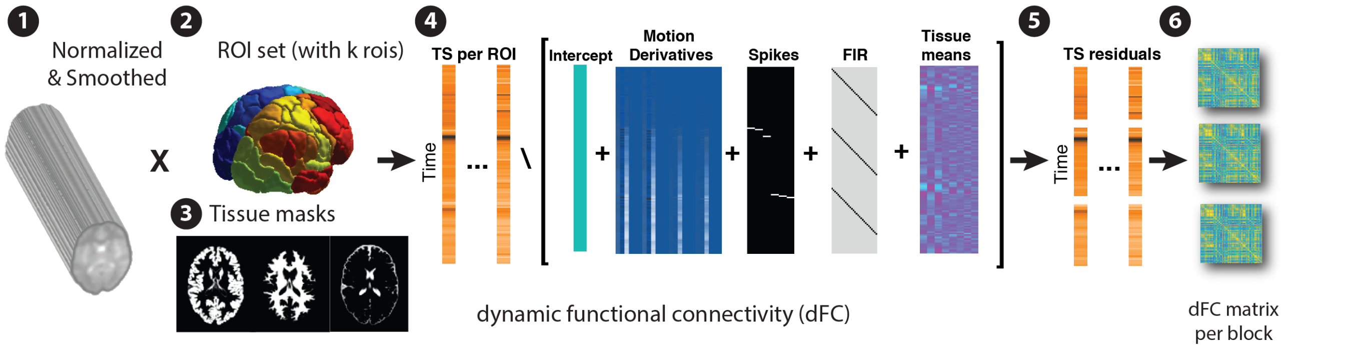

SUPPLEMENTARY FIGURE 6. FIR dFC estimation
(1) For each EPI 4D volume a temporal mask is defined as the voxels with standard deviation greater than zero. (2) Then all time-series (TS) within that mask are demeaned and regressed onto the ROI set to get one TS for each label. (3) Tissue specific volumes (thresholded >0.75) are used to create Tissue means and derivatives. (4) The ROI TS are demeaned and detrended to produce a TS data-stack, and a nuisance multi-regression model is used to regress out information of no interest. The nuisance variables are formed by an intercept, the Friston 24 motion derivatives, the frame wise displacement spike estimate, the stacked FIR matrix (addressing the 3 task block replications) and tissue means and first order derivatives. (5) TS residuals are calculated by subtracting the predicted TS from the original TS, and per block FIR dFC matrices are estimated using cross correlation.

The finite impulse response (FIR) dynamic functional connectivity estimation approach involves two distinct stages. In the first stage the regions of interest (ROI) set is used to extract mean time-series (TS) per ROI and the following multivariate regression model is used to regress out features of no interest:

$$Y = \beta_0 + [M, S, FIR, TM]\beta_G + \epsilon$$

Where Y are the ROI TS, M are the 24 motion derivatives, S are the motion spikes, FIR are the block FIR blocks and TM are the volume specific tissue and global means and their derivatives. In the second stage for each task individual block windows are extracted from the TS residuals and are then used to form the connectome using pairwise Pearson correlation.

C. Machine learning analysis

In this study we used both single metric models and stacked ones.

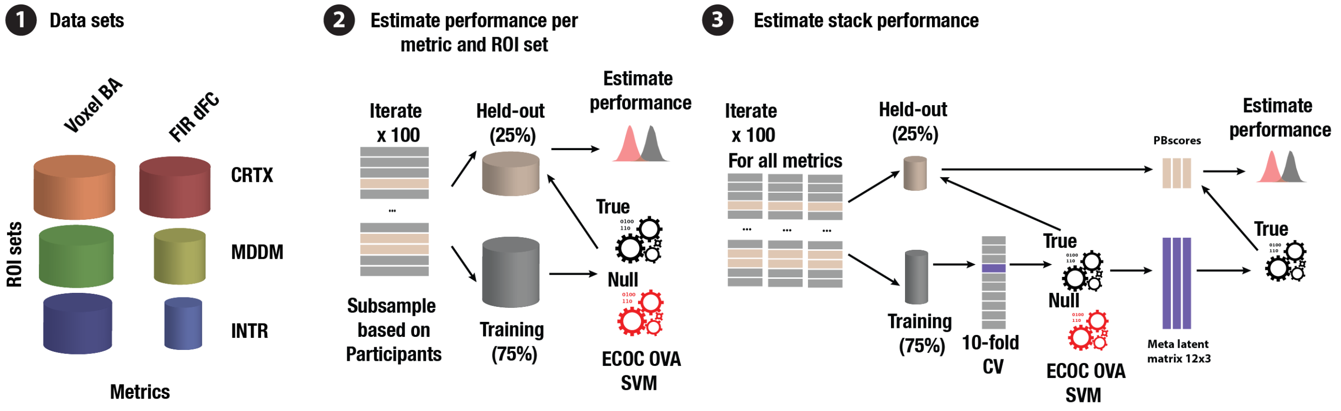

SUPPLEMENTARY FIGURE 7. (1) Input- fMRI data-set from 60 participants, performing 12 different tasks, with three one-minute repetitions separated by 20 seconds resting periods were used to estimate voxel-wise activity and dynamic functional connectivity across three different brain parcellations. (2) Classification- At the simplest level, we relied on random sampling from our participants to establish the performance distribution of a model. At the first step, we randomly selected 45 participants and trained two multiway classifiers, one trained with actual labels (training model) and one trained on shuffled labels (null model). Both models were tested on the remaining data from 15 held-out participants (test set). This process was repeated 100 times to form statistically valid permutation distributions. Significance of the model was calculated by comparing the mean test set performance to the entire null distribution. (3) Two-stage stack classification- extended the standard classification process by treating the different metrics as an ensemble of learners and training an additional model on all latent vectors across the per metric models.

D. Region of interest used in this study

This study includes three independent ROI sets,

SUPPLEMENTARY TABLE 2. INTR ROIs information

ID	Voxels	X	Y	Z	Hemisphere	lobe	RSN	Brodmann	AAL
1	525	-10	-96	0	Left	Occipital	VIS	BA.17	Calcarine L
2	1131	-28	-88	12	Left	Occipital	VIS	BA.19	Occipital Mid L
3	1395	32	-80	20	Right	Occipital	VIS	BA.19	Occipital Mid R
4	1042	-22	-64	52	Left	Parietal	DAN	BA.07	Parietal Sup L
5	1042	34	-70	-14	Right	Occipital	VIS	BA.19	Fusiform R
6	532	16	-94	4	Right	Occipital	VIS	BA.18	Calcarine R
7	1101	32	-56	48	Right	Parietal	DAN	BA.07	Parietal Sup R
8	1338	-36	-44	48	Left	Parietal	DAN	BA.40	Parietal Inf L
9	884	-20	-76	-18	Left	Cerebelum	VIS	BA.18	Cerebelum 6 L
10	483	14	-78	-18	Right	Cerebelum	VIS	BA.18	Cerebelum 6 R
11	301	22	-66	56	Right	Parietal	DAN	BA.07	Parietal Sup R
12	348	-24	-72	32	Left	Occipital	DAN	BA.07	Occipital Mid L
13	304	-42	-72	-4	Left	Occipital	VIS	BA.37	Occipital Mid L
14	404	28	-54	-22	Right	Cerebelum	SOM	BA.37	Cerebelum 6 R
15	546	48	8	32	Right	Frontal	DAN	BA.09	Frontal Inf Oper R
16	475	-46	2	34	Left	Frontal	DAN	BA.09	Precentral L
17	1191	-32	-10	56	Left	Frontal	DAN	BA.06	Precentral L
18	138	-32	-22	66	Left	Frontal	SOM	BA.06	Precentral L

SUPPLEMENTARY TABLE 2. INTR ROIs information

ID	Voxels	X	Y	Z	Hemisphere	lobe	RSN	Brodmann	AAL
19	522	2	10	52	Right	Frontal	VAN	BA.06	Supp Motor Area L
20	548	34	-2	54	Right	Frontal	DAN	BA.06	Frontal Sup 2 R

SUPPLEMENTARY TABLE 3. MDDM ROIs information

ID	Voxels	X	Y	Z	Hemisphere	lobe	RSN	Brodmann	AAL
1	665	46	-16	12	Right	Frontal	SOM	BA.13	Rolandic Oper R
2	109	50	-8	-24	Right	Temporal	DMN	BA.21	Temporal Mid R
3	143	40	-28	18	Right	Frontal	SOM	BA.13	Rolandic Oper R
4	1109	52	-10	-10	Right	Temporal	DMN	BA.21	Temporal Mid R
5	209	52	-30	16	Right	Temporal	SOM	BA.40	Temporal Sup R
6	137	46	8	-26	Right	Temporal	DMN	BA.38	Temporal Pole Sup R
7	651	-44	-16	12	Left	Frontal	SOM	BA.13	Rolandic Oper L
8	95	-48	-6	-24	Left	Temporal	DMN	BA.21	Temporal Mid L
9	154	-38	-26	18	Left	Frontal	SOM	BA.13	Rolandic Oper L
10	1126	-50	-10	-10	Left	Temporal	DMN	BA.21	Temporal Mid L
11	209	-50	-30	16	Left	Temporal	SOM	BA.40	Temporal Sup L
12	137	-44	8	-26	Left	Temporal	DMN	BA.38	Temporal Pole Sup L
13	230	8	52	30	Right	Frontal	DMN	BA.09	Frontal Sup Medial R
14	326	-4	52	30	Left	Frontal	DMN	BA.09	Frontal Sup Medial L
15	218	2	48	42	Right	Frontal	DMN	BA.09	Frontal Sup Medial L
16	287	18	34	48	Right	Frontal	DMN	BA.08	Frontal Sup 2 R
17	287	-16	34	48	Left	Frontal	DMN	BA.08	Frontal Sup 2 L
18	163	10	60	20	Right	Frontal	DMN	BA.10	Frontal Sup Medial R
19	157	-8	60	20	Left	Frontal	DMN	BA.10	Frontal Sup Medial L
20	855	6	50	2	Right	Frontal	DMN	BA.10	Frontal Sup Medial R
21	686	-6	50	2	Left	Frontal	DMN	BA.10	Frontal Sup Medial L
22	1426	0	34	-6	Medial	LIM	DMN	BA.32	Cingulate Ant L
23	52	0	36	-22	Medial	Frontal	LIM	BA.11	Rectus L
24	435	0	-26	56	Medial	Parietal	SOM	BA.06	Paracentral Lobule L
25	111	0	-12	50	Medial	Frontal	SOM	BA.06	Supp Motor Area L
26	1467	0	-40	36	Medial	LIM	DMN	BA.31	Cingulate Mid L
27	296	0	-12	42	Medial	LIM	SOM	BA.24	Cingulate Mid L
28	349	0	-60	26	Medial	Parietal	DMN	BA.31	Precuneus L
29	136	10	-54	16	Right	Parietal	DMN	BA.30	Precuneus R
30	136	-8	-54	16	Left	Parietal	DMN	BA.30	Precuneus L
31	249	46	-66	26	Right	Parietal	DMN	BA.39	Angular R
32	249	-44	-66	26	Left	Parietal	DMN	BA.39	Angular L
33	89	28	-58	-30	Right	Cerebelum	FPN	null	Cerebelum 6 R
34	89	-26	-58	-30	Left	Cerebelum	FPN	null	Cerebelum 6 L
35	400	0	-56	-20	Medial	Cerebelum	SOM	null	Vermis 4 5
36	3877	30	-56	46	Right	Parietal	DAN	BA.07	Parietal Sup R
37	2461	34	-72	-2	Right	Occipital	VIS	BA.19	Fusiform R
38	3877	-28	-56	46	Left	Parietal	DAN	BA.07	Parietal Inf L
39	2461	-32	-72	-2	Left	Occipital	VIS	BA.19	Occipital Mid L

SUPPLEMENTARY TABLE 3. MDDM ROIs information

ID	Voxels	X	Y	Z	Hemisphere	lobe	RSN	Brodmann	AAL
40	811	36	18	2	Right	Insula	VAN	BA.13	Insula R
41	1027	44	2	32	Right	Frontal	DAN	BA.09	Precentral R
42	916	28	-4	56	Right	Frontal	DAN	BA.06	Frontal Sup 2 R
43	811	-34	18	2	Left	Insula	VAN	BA.13	Insula L
44	1027	-42	2	32	Left	Frontal	DAN	BA.09	Precentral L
45	916	-26	-4	56	Left	Frontal	DAN	BA.06	Frontal Sup 2 L
46	436	14	-10	8	Right	subcorticalGM	FPN	null	Thalamus R
47	436	-12	-10	8	Left	subcorticalGM	FPN	null	Thalamus L
48	450	36	46	18	Right	Frontal	FPN	BA.10	Frontal Mid 2 R
49	570	42	32	26	Right	Frontal	FPN	BA.46	Frontal Mid 2 R
50	450	-34	46	18	Left	Frontal	FPN	BA.10	Frontal Mid 2 L
51	570	-40	32	26	Left	Frontal	FPN	BA.46	Frontal Mid 2 L
52	1890	0	14	44	Medial	Frontal	VAN	BA.32	Supp Motor Area L

SUPPLEMENTARY TABLE 4. CRTX ROIs, Numeric id, volume in voxels, MNI coordinates of their centre, Hemisphere, Lobe, Resting state network label, Brodmann area and the automatic anatomical label

ID	Voxels	X	Y	Z	Hemisphere	lobe	RSN	Brodmann	AAL
1	545	-22	-54	-10	Left	Occipital	VIS	BA.19	Lingual L
2	1127	-24	-78	-14	Left	Occipital	VIS	BA.18	Fusiform L
3	727	-44	-70	-10	Left	Occipital	VIS	BA.19	Occipital Inf L
4	517	-8	-68	-6	Left	Occipital	VIS	BA.18	Lingual L
5	765	-26	-96	-14	Left	Occipital	VIS	BA.18	Lingual L
6	163	-14	-46	-4	Left	Occipital	VIS	BA.30	Lingual L
7	835	-4	-94	-6	Left	Occipital	VIS	BA.18	Calcarine L
8	711	-46	-70	8	Left	Temporal	VIS	BA.39	Temporal Mid L
9	793	-22	-98	4	Left	Occipital	VIS	BA.18	Occipital Mid L
10	940	-10	-70	6	Left	Occipital	VIS	BA.30	Calcarine L
11	827	-38	-86	10	Left	Occipital	VIS	BA.19	Occipital Mid L
12	339	-12	-74	22	Left	Occipital	VIS	BA.31	Cuneus L
13	1138	-6	-88	26	Left	Occipital	VIS	BA.19	Cuneus L
14	787	-22	-88	24	Left	Occipital	VIS	BA.19	Occipital Sup L
15	593	-50	-6	-4	Left	Temporal	SOM	BA.22	Temporal Sup L
16	793	-52	-26	8	Left	Temporal	SOM	BA.41	Temporal Sup L
17	495	-36	-22	14	Left	Frontal	SOM	BA.13	Rolandic Oper L
18	648	-54	-6	10	Left	Frontal	SOM	BA.06	Rolandic Oper L
19	386	-52	-24	18	Left	Parietal	SOM	BA.13	Postcentral L
20	846	-56	-10	30	Left	Parietal	SOM	BA.06	Postcentral L
21	528	-46	-10	46	Left	Parietal	SOM	BA.06	Postcentral L
22	544	-6	-14	46	Left	Limbic	SOM	BA.24	Cingulate Mid L
23	432	-48	-30	56	Left	Parietal	SOM	BA.02	Postcentral L
24	548	-38	-24	54	Left	Parietal	SOM	BA.04	Postcentral L
25	369	-30	-48	62	Left	Parietal	SOM	BA.05	Parietal Sup L
26	619	-32	-22	62	Left	Frontal	SOM	BA.06	Precentral L
27	369	-26	-40	66	Left	Parietal	SOM	BA.03	Postcentral L
28	543	-20	-12	68	Left	Frontal	SOM	BA.06	Precentral L

SUPPLEMENTARY TABLE 4. CRTX ROIs, Numeric id, volume in voxels, MNI coordinates of their centre, Hemisphere, Lobe, Resting state network label, Brodmann area and the automatic anatomical label

ID	Voxels	X	Y	Z	Hemisphere	lobe	RSN	Brodmann	AAL
29	1090	-4	-32	66	Left	Parietal	SOM	BA.06	Paracentral Lobule L
30	290	-18	-32	66	Left	Parietal	SOM	BA.04	Postcentral L
31	1244	-42	-50	-20	Left	Temporal	DAN	BA.37	Temporal Inf L
32	631	-56	-62	-2	Left	Temporal	DAN	BA.37	Temporal Mid L
33	569	-24	-70	38	Left	Occipital	DAN	BA.07	Occipital Mid L
34	526	-54	-28	40	Left	Parietal	DAN	BA.02	Parietal Inf L
35	408	-40	-36	46	Left	Parietal	DAN	BA.40	Postcentral L
36	408	-32	-50	46	Left	Parietal	DAN	BA.40	Parietal Inf L
37	614	-16	-74	54	Left	Parietal	DAN	BA.07	Parietal Sup L
38	328	-28	-60	58	Left	Parietal	DAN	BA.07	Parietal Sup L
39	662	-4	-60	56	Left	Parietal	DAN	BA.07	Precuneus L
40	629	-16	-54	68	Left	Parietal	DAN	BA.07	Parietal Sup L
41	750	-30	-6	52	Left	Frontal	DAN	BA.06	Precentral L
42	586	-22	4	60	Left	Frontal	DAN	BA.06	Frontal Sup 2 L
43	414	-46	4	28	Left	Frontal	DAN	BA.09	Precentral L
44	576	-54	-40	20	Left	Temporal	VAN	BA.40	Temporal Sup L
45	695	-60	-26	28	Left	Parietal	VAN	BA.40	SupraMarginal L
46	534	-58	-40	36	Left	Parietal	VAN	BA.40	SupraMarginal L
47	706	-38	-4	-4	Left	Insula	VAN	BA.13	Insula L
48	469	-32	20	4	Left	Insula	VAN	BA.13	Insula L
49	292	-38	0	10	Left	Insula	VAN	BA.13	Insula L
50	495	-50	8	10	Left	Frontal	VAN	BA.44	Frontal Inf Oper L
51	1310	-28	42	30	Left	Frontal	VAN	BA.09	Frontal Mid 2 L
52	782	-4	8	40	Left	Limbic	VAN	BA.32	Cingulate Mid L
53	631	-10	-36	46	Left	Limbic	VAN	BA.31	Cingulate Mid L
54	524	-6	-4	64	Left	Frontal	VAN	BA.06	Supp Motor Area L
55	527	-22	20	-20	Left	Frontal	Limbic	BA.47	OFCpost L
56	1329	-8	34	-22	Left	Frontal	Limbic	BA.11	Rectus L
57	890	-28	-6	-40	Left	Occipital	Limbic	BA.20	Fusiform L
58	1023	-44	-22	-32	Left	Temporal	Limbic	BA.20	Temporal Inf L
59	610	-26	8	-36	Left	Temporal	Limbic	BA.38	Temporal Pole Mid L
60	370	-42	6	-20	Left	Temporal	Limbic	BA.38	Temporal Pole Sup L
61	455	-52	-52	44	Left	Parietal	FPN	BA.40	Parietal Inf L
62	647	-34	-64	46	Left	Parietal	FPN	BA.40	Parietal Inf L
63	430	-44	-42	46	Left	Parietal	FPN	BA.40	Parietal Inf L
64	696	-60	-44	-14	Left	Temporal	FPN	BA.21	Temporal Mid L
65	539	-30	42	-14	Left	Frontal	FPN	BA.11	OFCant L
66	607	-40	48	-6	Left	Frontal	FPN	BA.10	Frontal Mid 2 L
67	727	-26	58	8	Left	Frontal	FPN	BA.10	Frontal Sup 2 L
68	1260	-40	40	16	Left	Frontal	FPN	BA.46	Frontal Mid 2 L
69	829	-44	20	26	Left	Frontal	FPN	BA.09	Frontal Inf Tri L
70	671	-42	6	42	Left	Frontal	FPN	BA.06	Precentral L
71	527	-8	-74	36	Left	Parietal	FPN	BA.07	Precuneus L
72	208	-4	-30	26	Left	Limbic	FPN	BA.23	Cingulate Post L

SUPPLEMENTARY TABLE 4. CRTX ROIs, Numeric id, volume in voxels, MNI coordinates of their centre, Hemisphere, Lobe, Resting state network label, Brodmann area and the automatic anatomical label

ID	Voxels	X	Y	Z	Hemisphere	lobe	RSN	Brodmann	AAL
73	164	-4	4	28	Left	Limbic	FPN	BA.24	Cingulate Ant L
74	1237	-46	8	-34	Left	Temporal	DMN	BA.38	Temporal Inf L
75	777	-60	-20	-24	Left	Temporal	DMN	BA.20	Temporal Mid L
76	942	-56	-6	-14	Left	Temporal	DMN	BA.21	Temporal Mid L
77	787	-56	-32	-4	Left	Temporal	DMN	BA.21	Temporal Mid L
78	806	-58	-42	6	Left	Temporal	DMN	BA.22	Temporal Mid L
79	547	-48	-58	16	Left	Temporal	DMN	BA.39	Temporal Mid L
80	626	-38	-80	30	Left	Occipital	DAN	BA.19	Occipital Mid L
81	391	-56	-54	28	Left	Parietal	DMN	BA.40	SupraMarginal L
82	1073	-44	-66	38	Left	Parietal	DMN	BA.39	Angular L
83	585	-34	20	-14	Left	Insula	DMN	BA.47	Insula L
84	557	-4	34	-10	Left	Frontal	DMN	BA.32	Frontal Med Orb L
85	438	-44	30	-8	Left	Frontal	DMN	BA.47	Frontal Inf Orb 2 L
86	1093	-12	62	-6	Left	Frontal	DMN	BA.10	Frontal Sup 2 L
87	842	-50	22	6	Left	Frontal	DMN	BA.45	Frontal Inf Tri L
88	623	-6	44	6	Left	Limbic	DMN	BA.32	Cingulate Ant L
89	1015	-8	58	18	Left	Frontal	DMN	BA.10	Frontal Sup Medial L
90	607	-4	30	24	Left	Limbic	DMN	BA.32	Cingulate Ant L
91	730	-10	46	44	Left	Frontal	DMN	BA.08	Frontal Sup 2 L
92	689	-2	32	42	Left	Frontal	DMN	BA.08	Frontal Sup Medial L
93	680	-38	18	48	Left	Frontal	DMN	BA.08	Frontal Mid 2 L
94	861	-22	24	48	Left	Frontal	DMN	BA.08	Frontal Sup 2 L
95	686	-8	16	62	Left	Frontal	DMN	BA.06	Supp Motor Area L
96	539	-10	-56	12	Left	Occipital	DMN	BA.30	Calcarine L
97	702	-4	-56	26	Left	Parietal	DMN	BA.31	Precuneus L
98	429	-2	-30	36	Left	Limbic	DMN	BA.31	Cingulate Mid L
99	588	-6	-56	40	Left	Parietal	DMN	BA.07	Precuneus L
100	692	-24	-32	-18	Left	Limbic	VIS	BA.36	ParaHippocampal L
101	798	40	-36	-24	Right	Occipital	VIS	BA.20	Fusiform R
102	676	28	-36	-16	Right	Limbic	VIS	BA.36	ParaHippocampal R
103	1288	30	-70	-14	Right	Occipital	VIS	BA.19	Fusiform R
104	659	14	-66	-6	Right	Occipital	VIS	BA.19	Lingual R
105	684	50	-72	-8	Right	Temporal	VIS	BA.19	Temporal Inf R
106	993	12	-94	-6	Right	Occipital	VIS	BA.18	Lingual R
107	377	18	-48	-2	Right	Occipital	VIS	BA.30	Lingual R
108	1027	32	-94	-4	Right	Occipital	VIS	BA.18	Occipital Inf R
109	759	10	-76	8	Right	Occipital	VIS	BA.18	Calcarine R
110	385	22	-60	6	Right	Occipital	VIS	BA.30	Calcarine R
111	680	44	-80	8	Right	Occipital	VIS	BA.19	Occipital Mid R
112	1198	20	-92	20	Right	Occipital	VIS	BA.19	Occipital Sup R
113	683	12	-74	24	Right	Occipital	VIS	BA.31	Cuneus R
114	461	18	-86	38	Right	Occipital	VIS	BA.19	Cuneus R
115	814	34	-76	30	Right	Occipital	VIS	BA.19	Occipital Mid R
116	602	52	-16	4	Right	Temporal	SOM	BA.22	Temporal Sup R

SUPPLEMENTARY TABLE 4. CRTX ROIs, Numeric id, volume in voxels, MNI coordinates of their centre, Hemisphere, Lobe, Resting state network label, Brodmann area and the automatic anatomical label

ID	Voxels	X	Y	Z	Hemisphere	lobe	RSN	Brodmann	AAL
117	530	64	-24	6	Right	Temporal	SOM	BA.42	Temporal Sup R
118	382	40	-14	14	Right	Insula	SOM	BA.13	Insula R
119	398	46	-28	18	Right	Frontal	SOM	BA.13	Rolandic Oper R
120	279	60	0	10	Right	Frontal	SOM	BA.06	Rolandic Oper R
121	294	58	-12	14	Right	Frontal	SOM	BA.43	Rolandic Oper R
122	771	58	-6	30	Right	Parietal	SOM	BA.06	Postcentral R
123	239	10	-16	40	Right	Limbic	SOM	BA.24	Cingulate Mid R
124	466	52	-24	50	Right	Parietal	SOM	BA.03	Postcentral R
125	399	48	-12	48	Right	Frontal	SOM	BA.06	Precentral R
126	465	8	-12	50	Right	Frontal	SOM	BA.06	Supp Motor Area R
127	357	40	-24	56	Right	Frontal	SOM	BA.04	Precentral R
128	276	32	-42	62	Right	Parietal	SOM	BA.40	Postcentral R
129	528	32	-22	64	Right	Frontal	SOM	BA.06	Precentral R
130	282	32	-36	64	Right	Parietal	SOM	BA.03	Postcentral R
131	655	24	-10	66	Right	Frontal	SOM	BA.06	Frontal Sup 2 R
132	375	12	-40	68	Right	Parietal	SOM	BA.05	Paracentral Lobule R
133	692	8	-24	66	Right	Frontal	SOM	BA.06	Supp Motor Area R
134	248	22	-30	70	Right	Frontal	SOM	BA.04	Precentral R
135	1196	52	-54	-16	Right	Temporal	DAN	BA.37	Temporal Inf R
136	974	52	-60	8	Right	Temporal	DAN	BA.39	Temporal Mid R
137	377	60	-18	34	Right	Parietal	DAN	BA.04	Postcentral R
138	453	48	-38	48	Right	Parietal	DAN	BA.40	Parietal Inf R
139	424	42	-32	46	Right	Parietal	DAN	BA.40	Postcentral R
140	459	16	-74	52	Right	Parietal	DAN	BA.07	Parietal Sup R
141	555	36	-48	50	Right	Parietal	DAN	BA.40	Parietal Inf R
142	855	28	-62	56	Right	Parietal	DAN	BA.07	Parietal Sup R
143	733	10	-56	60	Right	Parietal	DAN	BA.07	Precuneus R
144	605	22	-50	70	Right	Parietal	DAN	BA.07	Parietal Sup R
145	505	36	-6	52	Right	Frontal	DAN	BA.06	Precentral R
146	633	26	6	56	Right	Frontal	DAN	BA.06	Frontal Sup 2 R
147	823	52	10	20	Right	Frontal	DAN	BA.44	Frontal Inf Oper R
148	491	58	-46	8	Right	Temporal	VAN	BA.22	Temporal Mid R
149	392	62	-40	16	Right	Temporal	VAN	BA.22	Temporal Sup R
150	952	62	-28	26	Right	Parietal	VAN	BA.40	SupraMarginal R
151	409	52	2	40	Right	Frontal	VAN	BA.06	Precentral R
152	367	42	4	-16	Right	Temporal	VAN	BA.38	Temporal Pole Sup R
153	752	48	-4	-6	Right	Insula	SOM	BA.22	Insula R
154	364	38	22	4	Right	Insula	VAN	BA.13	Insula R
155	890	44	6	2	Right	Insula	VAN	BA.13	Insula R
156	727	8	8	40	Right	Limbic	VAN	BA.32	Cingulate Mid R
157	587	12	-36	46	Right	Limbic	VAN	BA.31	Cingulate Mid R
158	608	10	2	64	Right	Frontal	VAN	BA.06	Supp Motor Area R
159	854	12	38	-22	Right	Frontal	Limbic	BA.11	OFCmed R
160	739	30	22	-20	Right	Frontal	Limbic	BA.47	OFCpost R

SUPPLEMENTARY TABLE 4. CRTX ROIs, Numeric id, volume in voxels, MNI coordinates of their centre, Hemisphere, Lobe, Resting state network label, Brodmann area and the automatic anatomical label

ID	Voxels	X	Y	Z	Hemisphere	lobe	RSN	Brodmann	AAL
161	793	16	64	-8	Right	Frontal	Limbic	BA.10	Frontal Sup 2 R
162	1144	30	8	-38	Right	Temporal	Limbic	BA.38	Temporal Pole Mid R
163	1136	48	-14	-36	Right	Temporal	Limbic	BA.20	Temporal Inf R
164	544	26	-12	-32	Right	Limbic	Limbic	BA.35	ParaHippocampal R
165	433	64	-38	36	Right	Parietal	FPN	BA.40	SupraMarginal R
166	410	54	-42	48	Right	Parietal	FPN	BA.40	Parietal Inf R
167	756	38	-64	46	Right	Parietal	FPN	BA.40	Angular R
168	753	64	-42	-12	Right	Temporal	FPN	BA.21	Temporal Mid R
169	242	34	20	-10	Right	Insula	FPN	BA.47	Insula R
170	986	36	46	-14	Right	Frontal	FPN	BA.11	OFCant R
171	796	30	58	4	Right	Frontal	FPN	BA.10	Frontal Sup 2 R
172	1022	44	44	10	Right	Frontal	FPN	BA.10	Frontal Mid 2 R
173	1085	46	22	24	Right	Frontal	FPN	BA.46	Frontal Inf Tri R
174	1027	30	48	26	Right	Frontal	FPN	BA.10	Frontal Mid 2 R
175	448	42	32	36	Right	Frontal	FPN	BA.09	Frontal Mid 2 R
176	1103	44	14	48	Right	Frontal	FPN	BA.08	Frontal Mid 2 R
177	478	16	-70	36	Right	Parietal	FPN	BA.07	Precuneus R
178	340	6	-26	30	Right	Limbic	FPN	BA.23	Cingulate Mid R
179	210	6	2	28	Right	Limbic	FPN	BA.24	Cingulate Mid R
180	636	8	30	26	Right	Limbic	FPN	BA.32	Cingulate Ant R
181	824	8	24	54	Right	Frontal	FPN	BA.08	Supp Motor Area R
182	791	48	-70	26	Right	Parietal	DMN	BA.39	Angular R
183	855	56	-52	28	Right	Parietal	DMN	BA.40	Angular R
184	689	52	-60	44	Right	Parietal	DMN	BA.40	Angular R
185	1059	48	12	-30	Right	Temporal	DMN	BA.38	Temporal Pole Mid R
186	1134	62	-14	-22	Right	Temporal	DMN	BA.21	Temporal Mid R
187	712	56	-8	-10	Right	Temporal	DMN	BA.21	Temporal Sup R
188	476	64	-28	-6	Right	Temporal	DMN	BA.21	Temporal Mid R
189	465	54	-32	0	Right	Temporal	SOM	BA.22	Temporal Sup R
190	1043	52	26	0	Right	Frontal	DMN	BA.47	Frontal Inf Tri R
191	990	6	36	-16	Right	Frontal	DMN	BA.11	Frontal Med Orb R
192	591	8	40	4	Right	Limbic	DMN	BA.32	Cingulate Ant R
193	114	6	28	14	Right	Limbic	DMN	BA.24	Cingulate Ant R
194	1446	10	56	18	Right	Frontal	DMN	BA.10	Frontal Sup Medial R
195	933	16	46	42	Right	Frontal	DMN	BA.08	Frontal Sup 2 R
196	537	30	28	42	Right	Frontal	DMN	BA.08	Frontal Mid 2 R
197	468	24	24	52	Right	Frontal	DMN	BA.08	Frontal Sup 2 R
198	484	14	-56	14	Right	Parietal	DMN	BA.29	Precuneus R
199	924	8	-50	30	Right	Parietal	DMN	BA.31	Precuneus R
200	570	8	-60	44	Right	Parietal	DMN	BA.07	Precuneus R

E. Individual differences outlier analysis

We used minimum covariance determinant estimate to examine the individual overall classification accuracy of the tasks, and identify possible outliers.

SUPPLEMENTARY TABLE 5. Individual accuracy across metrics and ROI sets

Subject	Events	CRTX			MDDM			INTR			Outlier
		BA	dFC	Stack	BA	dFC	Stack	BA	dFC	Stack	
01AR	36	66.7	69.4	86.1	69.4	30.6	69.4	47.2	58.3	66.7	false
01GI	36	55.6	75.0	77.8	36.1	27.8	47.2	38.9	33.3	33.3	false
01IB	36	36.1	80.6	75.0	38.9	47.2	47.2	16.7	50.0	58.3	false
02ZP	36	38.9	44.4	55.6	50.0	27.8	44.4	38.9	47.2	52.8	false
03RI	36	75.0	77.8	77.8	52.8	47.2	66.7	36.1	50.0	63.9	false
04GG	36	36.1	66.7	63.9	36.1	36.1	47.2	47.2	44.4	38.9	false
06JV	36	58.3	86.1	91.7	50.0	47.2	61.1	50.0	47.2	72.2	false
06RO	36	47.2	72.2	86.1	38.9	27.8	44.4	36.1	44.4	38.9	false
07VI	36	55.6	80.6	75.0	50.0	41.7	50.0	38.9	47.2	47.2	false
08BV	36	52.8	69.4	75.0	38.9	47.2	58.3	47.2	22.2	52.8	false
08JO	36	69.4	80.6	97.2	61.1	41.7	83.3	61.1	55.6	66.7	false
08ZB	36	47.2	69.4	72.2	25.0	38.9	47.2	38.9	33.3	63.9	false
09JR	36	38.9	52.8	61.1	47.2	38.9	61.1	36.1	33.3	61.1	false
09ML	36	38.9	86.1	91.7	44.4	58.3	58.3	27.8	69.4	55.6	false
10BG	36	58.3	50.0	61.1	50.0	47.2	58.3	52.8	50.0	75.0	false
10JO	36	55.6	61.1	77.8	55.6	44.4	55.6	41.7	41.7	44.4	false
10RL	36	25.0	61.1	75.0	27.8	30.6	33.3	27.8	38.9	33.3	false
14CR	36	44.4	88.9	97.2	38.9	52.8	66.7	33.3	55.6	72.2	false
14GI	36	72.2	88.9	88.9	41.7	44.4	58.3	44.4	41.7	50.0	false
14LL	36	47.2	63.9	63.9	41.7	25.0	52.8	25.0	33.3	50.0	false
15GL	36	80.6	83.3	94.4	75.0	61.1	86.1	66.7	66.7	83.3	false
15IJ	36	55.6	77.8	77.8	52.8	63.9	69.4	30.6	44.4	38.9	false
15RR	36	16.7	69.4	69.4	22.2	27.8	41.7	11.1	16.7	11.1	false
15SB	36	38.9	55.6	55.6	27.8	13.9	33.3	33.3	27.8	30.6	false
16IC	36	47.2	94.4	86.1	52.8	75.0	69.4	22.2	63.9	38.9	false
16QS	36	52.8	72.2	75.0	50.0	36.1	61.1	33.3	75.0	69.4	false
16RM	36	38.9	61.1	63.9	38.9	27.8	50.0	41.7	44.4	47.2	false
17DM	36	63.9	86.1	94.4	41.7	63.9	72.2	44.4	55.6	75.0	false
17MB	36	58.3	88.9	86.1	47.2	63.9	66.7	55.6	63.9	63.9	false
17ZJ	36	36.1	50.0	55.6	33.3	19.4	41.7	19.4	16.7	38.9	false
21RS	36	77.8	88.9	94.4	52.8	50.0	77.8	33.3	61.1	72.2	false
21SC	36	30.6	61.1	58.3	33.3	47.2	47.2	30.6	38.9	41.7	false
22EJ	36	58.3	88.9	91.7	38.9	36.1	47.2	41.7	52.8	58.3	false
23CC	36	47.2	80.6	86.1	38.9	75.0	69.4	38.9	61.1	69.4	false
23RK	36	55.6	72.2	80.6	47.2	55.6	69.4	41.7	66.7	66.7	false
23RR	36	69.4	83.3	97.2	47.2	55.6	63.9	36.1	44.4	63.9	false
23ZY	36	52.8	66.7	72.2	50.0	41.7	55.6	27.8	61.1	61.1	false

SUPPLEMENTARY TABLE 5. Individual accuracy across metrics and ROI sets

Subject	Events	CRTX			MDDM			INTR			Outlier
		BA	dFC	Stack	BA	dFC	Stack	BA	dFC	Stack	
24RF	36	61.1	75.0	94.4	58.3	61.1	80.6	41.7	50.0	69.4	false
26AG	36	52.8	75.0	80.6	44.4	47.2	63.9	30.6	50.0	72.2	false
26BF	36	50.0	72.2	80.6	44.4	47.2	55.6	44.4	52.8	58.3	false
26IU	36	30.6	69.4	52.8	27.8	33.3	33.3	33.3	13.9	38.9	false
29MN	36	52.8	69.4	77.8	52.8	52.8	63.9	47.2	66.7	75.0	false
29RA	36	83.3	86.1	88.9	55.6	66.7	91.7	58.3	52.8	75.0	false
29XR	36	33.3	66.7	52.8	30.6	41.7	52.8	30.6	25.0	38.9	false
30RB	36	36.1	33.3	58.3	38.9	19.4	52.8	30.6	27.8	44.4	false
AQ21	36	63.9	86.1	88.9	52.8	66.7	72.2	41.7	44.4	55.6	false
BC25	36	30.6	30.6	44.4	30.6	19.4	27.8	19.4	19.4	13.9	false
BTH0	36	69.4	75.0	77.8	47.2	38.9	63.9	47.2	50.0	63.9	false
GZ09	36	50.0	80.6	83.3	36.1	50.0	66.7	33.3	52.8	52.8	false
HA28	36	58.3	80.6	72.2	36.1	52.8	61.1	52.8	47.2	61.1	false
II14	36	16.7	19.4	30.6	8.3	22.2	11.1	5.6	16.7	13.9	true
IY23	36	69.4	83.3	91.7	44.4	55.6	63.9	55.6	58.3	69.4	false
JF24	36	33.3	80.6	83.3	55.6	75.0	77.8	47.2	61.1	63.9	false
KK04	36	66.7	83.3	80.6	58.3	47.2	75.0	66.7	47.2	75.0	false
QZ04	36	63.9	91.7	91.7	61.1	61.1	69.4	44.4	55.6	63.9	false
TD30	36	47.2	80.6	80.6	36.1	47.2	50.0	50.0	50.0	58.3	false
TZ10	36	11.1	2.8	5.6	16.7	8.3	5.6	19.4	2.8	0.0	true
YA18	36	19.4	33.3	36.1	27.8	19.4	30.6	13.9	16.7	22.2	false
YN01	36	58.3	83.3	88.9	58.3	52.8	77.8	41.7	61.1	83.3	false
ZQ14	36	61.1	83.3	77.8	50.0	75.0	69.4	36.1	63.9	58.3	false

Data Availability

The raw data that supports the findings of this study is committed in the OpenNeuro(22) repository under https://openneuro.org/datasets/ds003148/versions/1.0.1 (23) The processed experimental data that supports the findings of this study is committed in figshare(24) under https://figshare.com/articles/dataset/Neuroimaging_evidence_for_a_network_sampling_theory_of_human_intelligence/13237316.

Code Availability

The MATLAB code used to create the analysis and figures is available online at https://github.com/esoreq/12_tasks_code.git.

References

1. Hampshire A, Highfield RR, Parkin BL, Owen AM (2012) Fractionating human intelligence. *Neuron* 76(6):1225–1237.
2. Gould RL, et al. (2005) Functional neuroanatomy of successful paired associate learning in alzheimer's disease. *American Journal of Psychiatry* 162(11):2049–2060.
3. Corsi P (1972) Memory and the medial temporal region of the brain. *Unpublished doctoral dissertation), McGill University, Montreal, QB.*
4. Inoue S, Matsuzawa T (2007) Working memory of numerals in chimpanzees. *Current Biology* 17(23):R1004–R1005.
5. Collins P, Roberts A, Dias R, Everitt B, Robbins T (1998) Perseveration and strategy in a novel spatial self-ordered sequencing task for nonhuman primates: effects of excitotoxic lesions and dopamine depletions of the prefrontal cortex. *Journal of cognitive neuroscience* 10(3):332–354.
6. Shallice T (1982) Specific impairments of planning. *Philosophical Transactions of the Royal Society of London. B, Biological Sciences* 298(1089):199–209.
7. Cattell RB (1940) A culture-free intelligence test. i. *Journal of Educational Psychology* 31(3):161.
8. Silverman I, et al. (2000) Evolved mechanisms underlying wayfinding: Further studies on the hunter-gatherer theory of spatial sex differences. *Evolution and Human Behavior* 21(3):201–213.
9. Treisman AM, Gelade G (1980) A feature-integration theory of attention. *Cognitive psychology* 12(1):97–136.
10. Folstein MF, Folstein SE, McHugh PR (1975) "mini-mental state": a practical method for grading the cognitive state of patients for the clinician. *Journal of psychiatric research* 12(3):189–198.
11. Stroop JR (1935) Studies of interference in serial verbal reactions. *Journal of experimental psychology* 18(6):643.
12. Baddeley AD (1968) A 3 min reasoning test based on grammatical transformation. *Psychonomic science* 10(10):341–342.
13. Wechsler D, De Lemos MM (1981) *Wechsler adult intelligence scale-revised.* (Harcourt Brace Jovanovich).
14. Kunic T, Wloka C, Tsotsos JK (2019) Smiler: Consistent and usable saliency model implementations. *2019 MODVIS Workshop.*
15. Zhao Q, Koch C (2013) Learning saliency-based visual attention: A review. *Signal Processing* 93(6):1401–1407.
16. Walther D, Koch C (2006) Modeling attention to salient proto-objects. *Neural networks* 19(9):1395–1407.
17. Hou X, Zhang L (2007) Saliency detection: A spectral residual approach in *2007 IEEE Conference on computer vision and pattern recognition.* (Ieee), pp. 1–8.
18. Ashburner J (2007) A fast diffeomorphic image registration algorithm. *Neuroimage* 38(1):95–113.
19. Yan CG, Craddock RC, He Y, Milham MP (2013) Addressing head motion dependencies for small-world topologies in functional connectomics. *Frontiers in human neuroscience* 7:910.
20. Friston KJ, Williams S, Howard R, Frackowiak RS, Turner R (1996) Movement-related effects in fmri time-series. *Magnetic resonance in medicine* 35(3):346–355.
21. Afyouni S, Nichols TE (2018) Insight and inference for dvars. *NeuroImage* 172:291–312.
22. Gorgolewski Kea (2017) Openneuro—a free online platform for sharing and analysis of neuroimaging data. *Organization for Human Brain Mapping. Vancouver, Canada* p. 1677.
23. Eyal Soreq AH (2020) Neuroimaging evidence for a network sampling theory of human intelligence.
24. Soreq E, Hampshire A, Daws RE, Violante I (2020) Neuroimaging evidence for a network sampling theory of human intelligence.
