# Supplementary material for: Neuroimaging evidence for a network sampling theory of individual differences in human intelligence test performance
Source: Nat Commun. 2021 Apr 6;12:2072. doi: 10.1038/s41467-021-22199-9 (PMC8024400; doi:10.1038/s41467-021-22199-9)
Supplement: Supplementary file 3 — Description of Additional Supplementary Files [file 41467_2021_22199_MOESM3_ESM.pdf]

Description of Additional Supplementary Files

File Name: Supplementary Movie 1.

Description: Paired Associates (PA) This task is based on a paradigm commonly used to assess memory impairments in ageing clinical populations. Boxes are displayed at random locations on an invisible 5*5 grid. The boxes open one after another to reveal an enclosed object. This is followed by displaying the objects in the centre of the grid in random order. The participant is required to click on the boxes that contained each object. The difficulty is adjusted by increasing/decreasing the number of object-box pairs by 1. Difficulty increases if all pairs are correctly recollected. Outcome measure is calculated as the maximum level achieved (max=24,min=2 Population (mean,sd) = 5.28±1.13). The supplementary movie shows on minute of task engagement shown in the middle panel. The top two panels show summary scores (normalized sum per frame) from two complementary visual saliency approximation algorithms. The bottom panels show approximated task engagement as dynamic heat-maps, where in the right panel we superimpose the static centre surround Saliency model over the task stimuli and in the right panel we show the dynamic spectral saliency model.

File Name: Supplementary Movie 2.

Description: Spatial Span (SS) Based on the Corsi Block Tapping Task, which is a classical task for measuring spatial short-term memory capacity. 16 squares are displayed in a 4*4 grid. A sub-set of these squares flashes in a random sequence (1 flash every 900 ms). The participant is required to repeat the sequence by clicking on the squares in the same order in which they were flashed. The difficulty is dynamically varied based on accuracy, such that if the sequence is performed correctly the length of the next sequence is increased by one flash, otherwise, the sequence is one flash shorter. Outcome measure is calculated as the maximum level achieved (max=16,min=2 Population (mean,sd) = 6.15±1.07). The supplementary movie shows on minute of task engagement shown in the middle panel. The top two panels show summary scores (normalized sum per frame) from two complementary visual saliency approximation algorithms. The bottom panels show approximated task engagement as dynamic heat-maps, where in the right panel we superimpose the static centre surround Saliency model over the task stimuli and in the right panel we show the dynamic spectral saliency model. 2

File Name: Supplementary Movie 3.

Description: Monkey Ladder (ML) Visuospatial working memory task based on the non-human primate literature. Sets of numbered squares are displayed at random locations within an invisible 5*5 grid. After a variable interval (900ms * number of squares), the numbers are removed while the squares are kept in the screen. Participants are required to click the squares in ascending numerical sequence. The difficulty of the task increased or decreased by 1 square depending on the accuracy of the response. Outcome measure is calculated as the maximum level achieved (max=25,min=2 population (mean,sd) = 7.85±1.154). The supplementary movie shows on minute of task engagement shown in the middle panel. The top two panels show summary scores (normalized sum per frame) from two complementary visual saliency approximation algorithms. The bottom panels show approximated task engagement as dynamic heat-maps, where in the right panel we

superimpose the static centre surround Saliency model over the task stimuli and in the right panel we show the dynamic spectral saliency model.

File Name: Supplementary Movie 4.

Description: Self-Ordered Search (SO) Based on a test used to measure strategy during search behaviour. Sets of boxes are displayed in random locations within an invisible 5*5 grid. The participant is required to find a hidden 'token' by clicking on a box at a time to reveal its contents. Once the token is found it is hidden in another box. On any given trial, the token is only placed in a box once, forcing the participant to search all boxes until the token has been found once in each box. If the participant clicks on the same box twice whilst looking for the token or searches a box in which the token has previously been found, this is an error and the trial ends. In this case, a new trial begins with one less box to search. If no errors are made, a new trial begins with one extra box. Outcome measure is calculated as the maximum level achieved (max=25,min=2 Population (mean,sd) = 8.23 ± 2.1). The supplementary movie shows on minute of task engagement shown in the middle panel. The top two panels show summary scores (normalized sum per frame) from two complementary visual saliency approximation algorithms. The bottom panels show approximated task engagement as dynamic heat-maps, where in the right panel we superimpose the static centre surround Saliency model over the task stimuli and in the right panel we show the dynamic spectral saliency model. 3

File Name: Supplementary Movie 5.

Description: Tree Task (TT) Spatial planning task based on the Tower of London Task, which is widely used to measure executive function. Beads with numbers are positioned on a tree-shaped frame. The participant is required to position the beads in ascending numerical order from left to right and top to bottom. The participant must solve as many trials as possible within the duration of the block. The difficulty is increased by increasing the number of beads and planning complexity in increasing steps. Trials are aborted if the participant makes more than twice the number of moves required to solve the problem. Outcome measures = total score. Population (mean,sd) = 64±10.185. The supplementary movie shows on minute of task engagement shown in the middle panel. The top two panels show summary scores (normalized sum per frame) from two complementary visual saliency approximation algorithms. The bottom panels show approximated task engagement as dynamic heat-maps, where in the right panel we superimpose the static centre surround Saliency model over the task stimuli and in the right panel we show the dynamic spectral saliency model.

File Name: Supplementary Movie 6.

Description: Odd one out (OO) A deductive reasoning task based on a sub-set of problems from the Cattell Culture Fair Intelligence Test. A 3*3 grid of cells is displayed on the screen, containing a varied number of copies of a particular shape. The features that make up the objects in each cell (colour, shape, number of copies) are related to each other according to a set of rules. The participant is required to deduce the rules that relate the object features and select the cell whose contents do not match the rules. If the sequence is correct the problem increases in complexity. Outcome measure = total correct. Population (mean,sd) = 10.43 ± 3.31. The supplementary movie shows on minute of task engagement shown in the middle panel. The top two panels show summary

scores (normalized sum per frame) from two complementary visual saliency approximation algorithms. The bottom panels show approximated task engagement as dynamic heat-maps, where in the right panel we superimpose the static centre surround Saliency model over the task stimuli and in the right panel we show the dynamic spectral saliency model. 4

File Name: Supplementary Movie 7.

Description: Spatial Rotation (RO) Tasks of this type are typically used to measure the ability to manipulate objects in mind. Two grids of coloured squares are displayed side by side rotated by a multiple of 90 degrees. When rotated, the grids are either identical or differ by the position of one square. The participant is asked to indicate whether the grids are identical. If the response is correct the number of squares increases and if it is incorrect the number of squares decreases. Outcome measure = total score. Population (mean,sd) = 88.72±36.32. The supplementary movie shows on minute of task engagement shown in the middle panel. The top two panels show summary scores (normalized sum per frame) from two complementary visual saliency approximation algorithms. The bottom panels show approximated task engagement as dynamic heat-maps, where in the right panel we superimpose the static centre surround Saliency model over the task stimuli and in the right panel we show the dynamic spectral saliency model.

File Name: Supplementary Movie 8.

Description: Feature Match (FM) Based on classic feature search tasks that have been historically used to measure attentional processing. Two grids are displayed, each containing a set of abstract shapes. In half of the trials, the grids differ by just one shape. The participant is required to indicate whether the grid's contents are identical. If a trial is correct the total number of shapes increases, if it is incorrect the number of shapes is reduced. Outcome measure = total score. Population (mean,sd) = 131.35 ± 32.79. The supplementary movie shows on minute of task engagement shown in the middle panel. The top two panels show summary scores (normalized sum per frame) from two complementary visual saliency approximation algorithms. The bottom panels show approximated task engagement as dynamic heat-maps, where in the right panel we superimpose the static centre surround Saliency model over the task stimuli and in the right panel we show the dynamic spectral saliency model. 5

File Name: Supplementary Movie 9.

Description: Interlocking Polygons (IP) Based on the Interlocking Pentagons task, which is often used in the assessment of age-related disorders. A pair of polygons is displayed on one side of the screen. The participant indicates whether a polygon displayed on the other side of the screen is identical to one of the interlocking polygons. If responses are correct the differences between polygons become increasingly subtle. If the response is incorrect the differences between polygons become more pronounced. Main outcome measure = total score. Population (mean,sd) = 51.41 ± 24.86. The supplementary movie shows on minute of task engagement shown in the middle panel. The top two panels show summary scores (normalized sum per frame) from two complementary visual saliency approximation algorithms. The bottom panels show approximated task engagement as dynamic heat-maps, where in the right panel we superimpose the static centre surround Saliency model over the task stimuli and in the right panel we show the dynamic spectral saliency model.

File Name: Supplementary Movie 10.

Description: Colour Word (CW) This is a more challenging variant on the Stroop test. A coloured word is displayed at the top of the screen. For example, the word RED drawn in blue ink. The participant indicates which of two coloured words at the bottom of the screen described the colour of the word at the top of the screen. The colour word mappings may be congruent, in-congruent, or doubly in-congruent, depending on whether the colour that a given word describes matches the colour of the ink. The participant solves as many problems as possible within the duration of the block. Outcome measure = total score. Population (mean,sd) = 30.92 ± 13.01. The supplementary movie shows on minute of task engagement shown in the middle panel. The top two panels show summary scores (normalized sum per frame) from two complementary visual saliency approximation algorithms. The bottom panels show approximated task engagement as dynamic heat-maps, where in the right panel we superimpose the static centre surround Saliency model over the task stimuli and in the right panel we show the dynamic spectral saliency model. 6

File Name: Supplementary Movie 11.

Description: Grammatical Reasoning (GR) This is a verbal reasoning task based on Alan Baddeley's 3-minute grammatical reasoning test. Problems of the form "The square is not encapsulated by the circle" are displayed on the screen and the participant indicates whether the statement correctly describes the pair of objects presented. Outcome measure is calculated as the maximum level achieved (max=25,min=2 Population (mean,sd) = 17.38 ± 5.01). The supplementary movie shows on minute of task engagement shown in the middle panel. The top two panels show summary scores (normalized sum per frame) from two complementary visual saliency approximation algorithms. The bottom panels show approximated task engagement as dynamic heat-maps, where in the right panel we superimpose the static centre surround Saliency model over the task stimuli and in the right panel we show the dynamic spectral saliency model.

File Name: Supplementary Movie 12.

Description: Digit Span (DS) Is a computerised variant on the verbal working memory component of the WAIS-R intelligence test. Participants view a sequence of digits that appear one after another. Subsequently, they repeat the sequence of numbers by clicking on the corresponding digit on a keyboard displayed on the screen. The difficulty is dynamically varied by increasing or decreasing the number of digits to remember by 1, depending on whether the participant got the previous trial correct. Outcome measure is calculated as the maximum level achieved (max=25,min=2 Population (mean,sd) = 7.22±1.52). The supplementary movie shows on minute of task engagement shown in the middle panel. The top two panels show summary scores (normalized sum per frame) from two complementary visual saliency approximation algorithms. The bottom panels show approximated task engagement as dynamic heat-maps, where in the right panel we superimpose the static centre surround Saliency model over the task stimuli and in the right panel we show the dynamic spectral saliency model. 7
