# Supplementary material for: Neuroimaging evidence for a network sampling theory of individual differences in human intelligence test performance
Source: Nat Commun. 2021 Apr 6;12:2072. doi: 10.1038/s41467-021-22199-9 (PMC8024400; doi:10.1038/s41467-021-22199-9)
Supplement: Supplementary file 16 — Reporting Summary [file 41467_2021_22199_MOESM16_ESM.pdf]

nature research

Corresponding author(s): Eyal Soreq

Last updated by author(s): Nov 30, 2020

Reporting Summary

Nature Research wishes to improve the reproducibility of the work that we publish. This form provides structure for consistency and transparency in reporting. For further information on Nature Research policies, see our Editorial Policies and the Editorial Policy Checklist.

Statistics

For all statistical analyses, confirm that the following items are present in the figure legend, table legend, main text, or Methods section.

n/a	Confirmed	
☐	☒	The exact sample size (*n*) for each experimental group/condition, given as a discrete number and unit of measurement
☐	☒	A statement on whether measurements were taken from distinct samples or whether the same sample was measured repeatedly
☐	☒	The statistical test(s) used AND whether they are one- or two-sided<br>*Only common tests should be described solely by name; describe more complex techniques in the Methods section.*
☐	☒	A description of all covariates tested
☐	☒	A description of any assumptions or corrections, such as tests of normality and adjustment for multiple comparisons
☐	☒	A full description of the statistical parameters including central tendency (e.g. means) or other basic estimates (e.g. regression coefficient) AND variation (e.g. standard deviation) or associated estimates of uncertainty (e.g. confidence intervals)
☐	☒	For null hypothesis testing, the test statistic (e.g. *F*, *t*, *r*) with confidence intervals, effect sizes, degrees of freedom and *P* value noted<br>*Give P values as exact values whenever suitable.*
☒	☐	For Bayesian analysis, information on the choice of priors and Markov chain Monte Carlo settings
☐	☒	For hierarchical and complex designs, identification of the appropriate level for tests and full reporting of outcomes
☒	☐	Estimates of effect sizes (e.g. Cohen's *d*, Pearson's *r*), indicating how they were calculated

Our web collection on statistics for biologists contains articles on many of the points above.

Software and code

Policy information about availability of computer code

Data collection	Individual tests for the imaging and Internet studies were programmed in the Adobe Flex development environment. In the imaging study, the tests ran as stand alone software within the AIR runtime environment. In the behavioural study, the tests were embedded in a custom-built web site programmed in Microsoft ASP.net.
Data analysis	Data analysis was performed using various versions of Matlab no older than 2016b. Machine learning analysis was conducted using the statistics and machine learning toolbox . Pre processing relied on SPM 12 FSL 5.01 and custom code written by the first author.

For manuscripts utilizing custom algorithms or software that are central to the research but not yet described in published literature, software must be made available to editors and reviewers. We strongly encourage code deposition in a community repository (e.g. GitHub). See the Nature Research guidelines for submitting code & software for further information.

Data

Policy information about availability of data

All manuscripts must include a data availability statement. This statement should provide the following information, where applicable:

- Accession codes, unique identifiers, or web links for publicly available datasets
- A list of figures that have associated raw data
- A description of any restrictions on data availability

The data that supports the findings of this study has been committed to the OpenNeuro repository under the accession code ds003093

Field-specific reporting

Please select the one below that is the best fit for your research. If you are not sure, read the appropriate sections before making your selection.

[x] Life sciences [ ] Behavioural & social sciences [ ] Ecological, evolutionary & environmental sciences

For a reference copy of the document with all sections, see nature.com/documents/nr-reporting-summary-flat.pdf

Life sciences study design

All studies must disclose on these points even when the disclosure is negative.

Sample size	Sixty adults (35 females, mean age 22.95, range 18-38 years of age). No formal power calculations were performed to determine sample size. However, we used previous cognitive fMRI experiments (Hampshire et al, Neuron, 2012; DOI:https://doi.org/10.1016/j.neuron.2012.06.022) as guidance for suitable sample sizes and we aimed to acquire a sample size larger than typically used in fMRI studies (approximately 20 subjects).
Data exclusions	Outlier detection methods were implemented at each stage of the analysis and all subjects were included at every step. However, at the level of performance index (first principal component across all behaviour measures) two participants were identified as outliers (using the robust multivariate covariance and mean estimate outlier detection using FAST-MCD (minimum covariance determinant)). These participants were thus excluded from this analysis.
Replication	This study employs machine learning. We report cross-validated out-of-sample results. We iteratively separated our data into two independent subject subsets (training = 75% and test = 25%), thus employing random sampling replication. The group comparisons rely on random permutation analysis, where the study events (36 per participant) are are split into a training (75%) and testing (25%) data subsets. Each of the subsets includes a unique sample of participants. For each analysis we repeat this process 100 times to generate statistical distribution.
Randomization	We used a within-subject design where all subjects were tested on the same experimental conditions. So randomizing participants to different conditions was not necessary.
Blinding	All participants were exposed to the same experimental conditions and were unaware of the purpose of the experiment. Thus blinding was not necessary.

Reporting for specific materials, systems and methods

We require information from authors about some types of materials, experimental systems and methods used in many studies. Here, indicate whether each material, system or method listed is relevant to your study. If you are not sure if a list item applies to your research, read the appropriate section before selecting a response.

Materials & experimental systems

n/a	Involved in the study	
[x]	[ ]	Antibodies
[x]	[ ]	Eukaryotic cell lines
[x]	[ ]	Palaeontology and archaeology
[x]	[ ]	Animals and other organisms
[ ]	[x]	Human research participants
[x]	[ ]	Clinical data
[x]	[ ]	Dual use research of concern

Methods

n/a	Involved in the study	
[x]	[ ]	ChIP-seq
[x]	[ ]	Flow cytometry
[ ]	[x]	MRI-based neuroimaging

Human research participants

Policy information about studies involving human research participants

Population characteristics	Sixty adults (35 females, mean age 22.95, range 18-38 years of age), all with normal hearing and corrected to normal vision were included in the study.
Recruitment	Participants were recruited from the University of Western Ontario and surrounding area. The participants chose to participate based on a brief description of the study. It is unlikely that this caused a significant bias that might impact the results.
Ethics oversight	This study was approved by the University of Western Ontario ethics committee and all participants provided written consent before taking part in the study.

Note that full information on the approval of the study protocol must also be provided in the manuscript.

Magnetic resonance imaging

Experimental design

Design type	block design
Design specifications	Each participant undertook twelve functional runs, one for each specific task. These were administered in a predefined order. Each experimental run contained three blocks each one-minute long, separated by 20s of rest.
Behavioral performance measures	All participants engaged with twelve cognitive tasks designed to measure planning, reasoning, attention, and working memory abilities that are believed to be core intelligence abilities. All tasks designs and behavioural scores are reported in detail in section 1.1 in the supplementary materials and movies. Before scanning, participants underwent a short training session to ensure that they could perform all 12 tasks. The training consisted of reading written instructions followed by one practice block of each task, undertaken on a laptop outside of the MRI scanner.

Acquisition

Imaging type(s)	Functional and structural
Field strength	3T
Sequence & imaging parameters	Images consisted of 36*3 mm slices, with an 80 x 80 matrix, 240 x 240 mm field of view, TE=30ms, flip angle=90 degree, echo spacing=2.65ms. A 1 mm resolution MPRAGE structural scan was also collected for each participant with a 256 x 240 x 384 matrix, TI=900ms, TR=2.3s, TE=2.98ms and 9 degree flip angle.
Area of acquisition	Whole brain

Diffusion MRI ☐ Used ☒ Not used

Preprocessing

Preprocessing software	SPM12 version = 6906,, FSL (FMRIB Software Library v5.0) and MATLAB 2016b
Normalization	All functional scans were non-linearly normalized onto MNI space using a DARTEL group template constructed from the structural scans of all individuals.
Normalization template	ICBM152
Noise and artifact removal	Both activation and connectivity rely on different noise removal strategies that are described in detail in the supplementary material
Volume censoring	Signal to noise ratio (SNR) metrics were extracted from the unprocessed fMRI images using an in-house implementation of the metrics proposed by Friedman\cite{friedman2006report}. An outlier's detection analysis was performed to detect low values (SNR <5). No scans were discarded at this stage.

Statistical modeling & inference

Model type and settings	Per subject 1st level design matrix was constructed using the classic mass-univariate GLM in SPM12. Predefined contrasts of interest, whole brain maps depicting statistical parametric estimates were generated. This was followed by a 2nd level group contrasts examining experimental factors as well as factor conjunctions
Effect(s) tested	Classification accuracy, performance index prediction and association between summary statistics

Specify type of analysis: ☐ Whole brain ☐ ROI-based ☒ Both

Anatomical location(s)	We used three different ROI atlases two of which were generated using data-driven algorithms developed by the first author. The third data set was an unbiased atlas generated using resting state functional data
Statistic type for inference (See Eklund et al. 2016)	Minimal cluster size was derived by performing uncorrected analysis with a relaxed threshold (P < 0.01), then we used the minimal false-discovery-rate (FDR) cluster (P_{FDR} < 0.05) to generate a cluster corrected map for each of the contrasts in the specific conjunction.
Correction	False-discovery-rate (FDR), permutation & monte carlo sampling were used in different parts of this study

Models & analysis

n/a	Involved in the study
☐	☒ Functional and/or effective connectivity
☐	☒ Graph analysis
☐	☒ Multivariate modeling or predictive analysis

Functional and/or effective connectivity	(1) For each EPI 4D volume a temporal mask is defined as the voxels with standard deviation greater than zero. (2) Then all time-series (TS) within that mask are demeaned and regressed onto the ROI set to get one TS for each label. (3) Tissue specific volumes (thresholded >0.75) are used to create Tissue means and derivatives. (4) The ROI TS are demeaned and detrended to produce a TS data-stack, and a nuisance multi-regression model is used to regress out information of no interest. The nuisance variables are formed by an intercept, the Friston 24 motion derivatives, the frame wise displacement spike estimate, the stacked FIR matrix (addressing the 3 task block replications) and tissue means and first order derivatives. (5) TS residuals are calculated by subtracting the predicted TS from the original TS, and per block FIR dFC matrices are estimated using cross correlation.
Graph analysis	Weighted undirected connectivity matrices were formed by averaging between the two pairs of estimation between two nodes.
Multivariate modeling and predictive analysis	For classification analysis we used task class per block as response vector (independent variables). To perform classification we used dense (multi-class support vector machine) models. For testing we used both subject specific permutation partitioning as well as 5-fold cross validation within the training set. We used F1-score to evaluate classification accuracy. And used bootstrapping to form F1 distributions for statistical evaluation. For the predictive analysis we used ensemble boosted regression trees trained on meta connectivity scores used to predict performance index.
